# Assessing Exchange-Correlation Functionals for Accurate Densities of Solids

**DOI:** 10.1021/acs.jctc.4c01042

**Published:** 2024-12-03

**Authors:** Ayoub Aouina, Pedro Borlido, Miguel A. L. Marques, Silvana Botti

**Affiliations:** † Research Center Future Energy Materials and Systems of the University Alliance Ruhr and Interdisciplinary Centre for Advanced Materials Simulation, Ruhr University Bochum, Universitätsstraße 150, D-44801 Bochum, Germany; ‡ Institut für Festkörpertheorie und -optik, Friedrich-Schiller-Universität Jena, D-07743 Jena, Germany; § European Theoretical Spectroscopy Facility, Sart Tilman, Liège B-4000, Belgium; ∥ CFisUC, Department of Physics, University of Coimbra, Rua Larga, Coimbra 3004-516, Portugal

## Abstract

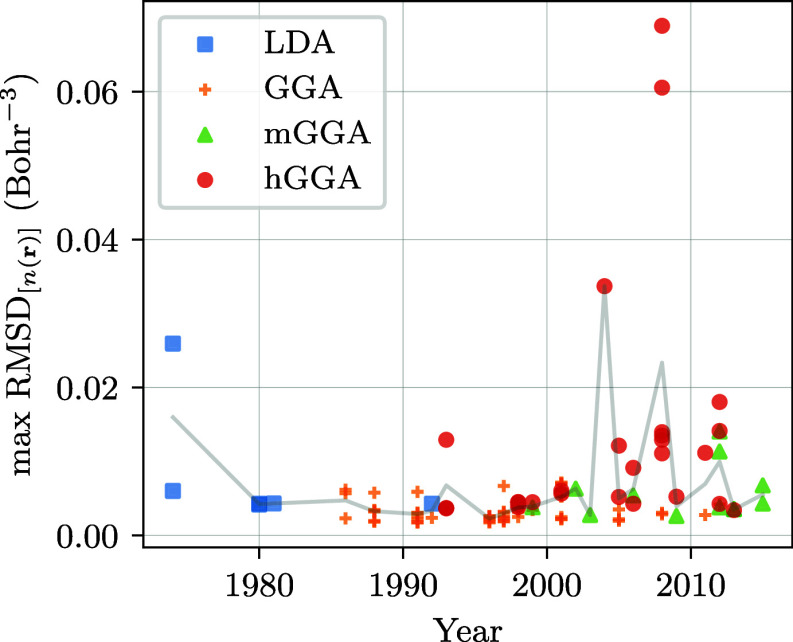

The success of Kohn–Sham density functional theory in predicting electronic properties from first-principles is key to its ubiquitous presence in condensed matter research. Central to this theory is the exchange-correlation functional, which can only be written in an approximate form using a handful of exact constraints. A recent criticism of these approximations is that they are designed to give an accurate description of the energy at the expense of a poor representation of the density, which is contrary to the spirit of density functional theory. These conclusions are drawn from studies of atoms or small molecules, where exact results are available. To shed light on this issue, we use the almost exact densities and energies of three prototypical solids (a semiconductor, silicon, an insulator, sodium chloride, and a metal, copper) to compare the performance of exchange-correlation functionals from all rungs of Jacob’s ladder. By examining their errors in reproducing both energy and density, we show that several hybrids and semilocal functionals perform consistently well. Furthermore, functionals built to reproduce exact constraints tend to be among the top performers for all tested material classes, strengthening the argument for using these constraints in functional construction. On average, functionals published up to the early 2000s simultaneously improve the prediction of both densities and energies. This is often not the case for more recent functionals, although errors in energy and density continue to evolve in a correlated manner.

## 1 Introduction

Among the various electronic structure methods available to the scientific community, density functional theory (DFT),^1^ and more specifically its Kohn–Sham formulation,^2^ has become the standard choice for the study of electronic systems such as atoms, molecules and solids.^3−7^ The DFT was formally established in 1964 with the Hohenberg–Kohn theorems.^1^ These theorems state that the ground-state density of a nonrelativistic system with a nondegenerate ground state uniquely determines the (static) external potential up to a constant. This implies that any observable of the system can be regarded as a functional of the ground-state density. Since this is also true for the energy, the variational principle becomes the recipe for finding the ground state. The original theorems were later generalized to other classes of ground states^8−12^ and Hamiltonians.^13−17^

To put this minimization procedure into a more practical framework, Kohn and Sham^2^ assumed the existence of an auxiliary system of independent particles with the same ground-state density as the real one. This ultimately leads to a set of single-particle integro-differential equations of the form
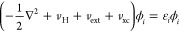
1Here, the analytic expressions of the Hartree potential
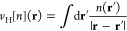
2(as a functional of the density *n*(**r**)) and of the external potential *v*_ext_ (usually of Coulombic origin) are known. The exchange-correlation (xc) potential *v*_xc_
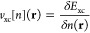
3defined as the functional derivative of an xc energy *E*_xc_, is however to a large extent unknown and requires appropriate approximations.

The popularity of the Kohn–Sham DFT has led to hundreds of different approximations for the xc functional,^18,19^ although only a few of them see widespread use in the community.^5,6^ Functionals are usually classified using the rungs of Jacob’s Ladder,^20^ which distinguishes them in terms of their ingredients but neglects other important aspects of their construction. The first four rungs of the ladder are the local density approximation (LDA), the generalized gradient approximation (GGA), the meta-GGA (mGGA), and the hybrid functionals (hGGA, constructed with occupied Kohn–Sham orbitals). We can also classify functionals in a spectrum ranging from fully ab initio functionals, which use only exact theoretical results in their construction, to fully empirical functionals, which rely only on numerical fits to experimental results. An example of a fully ab initio functional is the original LDA,^21,22^ while the Minnesota functionals^23^ are closer to the other end of the spectrum. We note that, in our view, functionals fitted to purely theoretical data should be considered fully ab initio, even if they contain a large number of parameters, as in the case of recent functionals based on machine learning. In general, we can expect that functionals constructed to obey exact theoretical constraints will be more transferable than functionals with many fitted parameters.

Three important quantities appear in Kohn–Sham DFT: the xc energy, the electronic density, and the xc potential. All three are related by construction, therefore one could expect that selectively improving one would lead to improvements also in the others. However, this does not always turn out to be true: in several examples^24,25^ it has been shown that density functionals yielding accurate energies can ironically produce unreliable and even unphysical densities and potentials. Most of our knowledge in this respect comes from calculations for small finite systems, such as atoms, ions and small molecules, thanks to the abundance of nearly exact quantum chemical data, while very little is known for extended systems.

Recently, Chen et al.^26^ computed accurate charge densities for extended systems (namely silicon, sodium chloride and copper), using auxiliary-field quantum Monte Carlo (QMC) methods,^27,28^ while at the same time comparing the performance of five approximations to the xc functional. This method is based on second quantization and uses imaginary-time evolution to propagate a trial wave function toward the ground state. It uses a stochastic approach to solve many-body quantum systems by transforming the two-body interaction problem into a statistical sampling of auxiliary fields. This method can handle pseudopotentials without requiring additional approximations, making the comparison with DFT results straightforward. Moreover, auxiliary field QMC allows the computation of properties beyond total energy, such as the charge density, through back-propagation.

In this work, we extend on this benchmark by comparing the density produced by a much larger set of 98 DFT functionals with the almost exact QMC results. This set includes LDA, GGA, mGGA, and hGGA functionals available via the Libxc library.^18,19^ The following of this paper is organized as follows. In Section 2 we present the general discussion of the data analysis and calculation details. In Section 7 we present the results of our calculations, alongside discussion on the obtained results. In Section 13 we offer our concluding remarks.

## 2 Methods

### 2.1 QMC Data

All QMC data (including densities and their respective uncertainties) were obtained from the repository of the original QMC paper,^29^ while the QMC values for the total energies are −217.05569 ± 0.00246 eV for Si, −1549.62405 ± 0.00714 eV for NaCl and −19176.63639 ± 0.14488 eV for Cu.^30^

### 2.2 DFT Calculations

All DFT calculations are spin unpolarized and were performed using the PWscf code of the Quantum ESPRESSO distribution,^31,32^ with xc functionals from the Libxc library.^18,19^ To ensure compatibility with the QMC data from ref (26). we used the same calculation parameters as there. We employed the same multiple-projector optimized norm-conserving pseudopotentials^29^ (ONCVPSP) generated according to the method of Hamann^33^ without nonlinear core corrections. Additionally, we used 6 × 6 × 6 k-point grids for Brillouin zone sampling, consistent with the choice made in the QMC study, where this grid was used to obtain the trial energy and wave function. This choice leads in most cases to a convergence error on the total energy per atom below 10 meV.

However, some meta-GGA functionals are known to show very slow convergence due to numerical instabilities.^34^ For this reason, the selected convergence parameters do not guarantee the same convergence error (in this case the error is ten times larger) for a small subgroup of functionals (including M06L, SCAN, revTPSS and PKZB). We remark that, according to our tests, the revised version r2SCAN does not resolve this issue.

We have also performed Hartree–Fock (HF) calculations for comparison using the same convergence parameters. In this case the convergence error (about 10 eV per atom) is significantly larger than our threshold and we decided therefore not to include these calculations. Qualitatively, we have observed much larger errors than for DFT functionals both for the energy and the density.

For Si the 2-atom primitive unit cell was used together with the conventional cell lattice parameter of 10.263087 Bohr. The pseudopotential was generated using Ne-shells as core states and considering 3s, 3p and 3d orbitals as valence. We set a plane wave cutoff of 25 Ry. For NaCl, the primitive cell of the rock salt structure was used together with a conventional lattice parameter of 10.7563 Bohr. Concerning the pseudopotentials, a [Ne] core was defined for Cl and a [He] core for Na. The plane wave cutoff was set to 40 Ry. Finally, for Cu we used the conventional 4-atom cubic cell with a lattice constant of 6.790 Bohr. The pseudopotential was similarly generated with a [Ne] core and we set a kinetic energy cutoff of 64 Ry. As Cu is a metal, we used Gaussian smearing with a broadening value of 0.001 Ry.

### 2.3 Error Analysis

To quantify the error of DFT quantities, we used three different descriptors. As in ref (25), using the density obtained from solving the Kohn–Sham equations, *n*^DFT^(**r**), we calculated its root-mean-square difference (RMSD) with respect to the QMC one, *n*^QMC^(**r**), as the integrated difference over the unit cell, i.e.
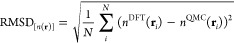
4with *N* being the number of grid points used in both calculations, determined by the kinetic energy cutoff. As a reference, the top 24 functionals for RMSD_[*n*(**r**)]_ show absolute errors in the range of 0.1 × 10^–3^–0.5 × 10^–3^ Bohr^–3^ for Si and NaCl, while Cu shows errors an order of magnitude larger.

With respect to the energy, we measure the deviations via the absolute error (AE),
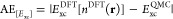
5Here, *E*_xc_^QMC^ is the QMC reference xc energy.^30^

Since the quantities RMSD_[*n*(**r**)]_ and AE_[*E*_xc_]_ have different units and orders of magnitude, we resort to a normalization scheme in order to ease comparison. Specifically, we normalize each of these quantities (for each material) with respect to their respective median, following the procedure of ref (25). The normalized quantities have the prefix “N”. It should be noted that other metrics have been proposed in the literature^35−37^ to assess the performance of the density functionals, among which we considered the normalized integral absolute deviation (see SI for further details). However, since this metric does not change the overall ranking of the functionals, we chose to primary use the RMSD_[*n*(**r**)]_ in our analysis.

We also employ Kendall’s τ coefficient^38^ to measure the ordinal correlation between the functionals performance for the energy and the density.

### 2.4 Kohn–Sham Inversion

In order to obtain *E*_xc_^QMC^, we must extract it from the QMC data. This can be done by inverting the Kohn–Sham energy, as
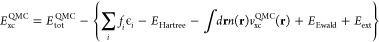
6where *f*_*i*_ denotes the occupation number, *E*_Ewald_ is the ion–ion electrostatic energy (also called Ewald term), *E*_ext_ is the contribution of the external potential to the total energy, and *E*_Hartree_ is the well-known Hartree energy,
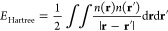
7The remaining terms involve *v*_xc_^QMC^, the xc functional that yields the QMC density, and {ϵ_*i*_}, the set of eigenvalues of the Kohn–Sham Hamiltonian built from *v*_xc_^QMC^. In order to obtain these quantities it is necessary to invert the Kohn–Sham equations at the fixed density *n*^QMC^. For this, we use the method of ref (39), which has proven very effective in the past.

## 3 Results and Discussion

We are now ready to compare and discuss the errors in the energy and density computed with 98 approximate xc functionals. A list of the considered functionals, indicating the corresponding rungs of the Jacob’s ladder, is given in the Supporting Information. We present results for three crystalline materials, Si, NaCl and Cu, as prototypical examples of covalent solids, ionic solids and metals,^40^ respectively.

### 3.1 Si

**Figure 1:**
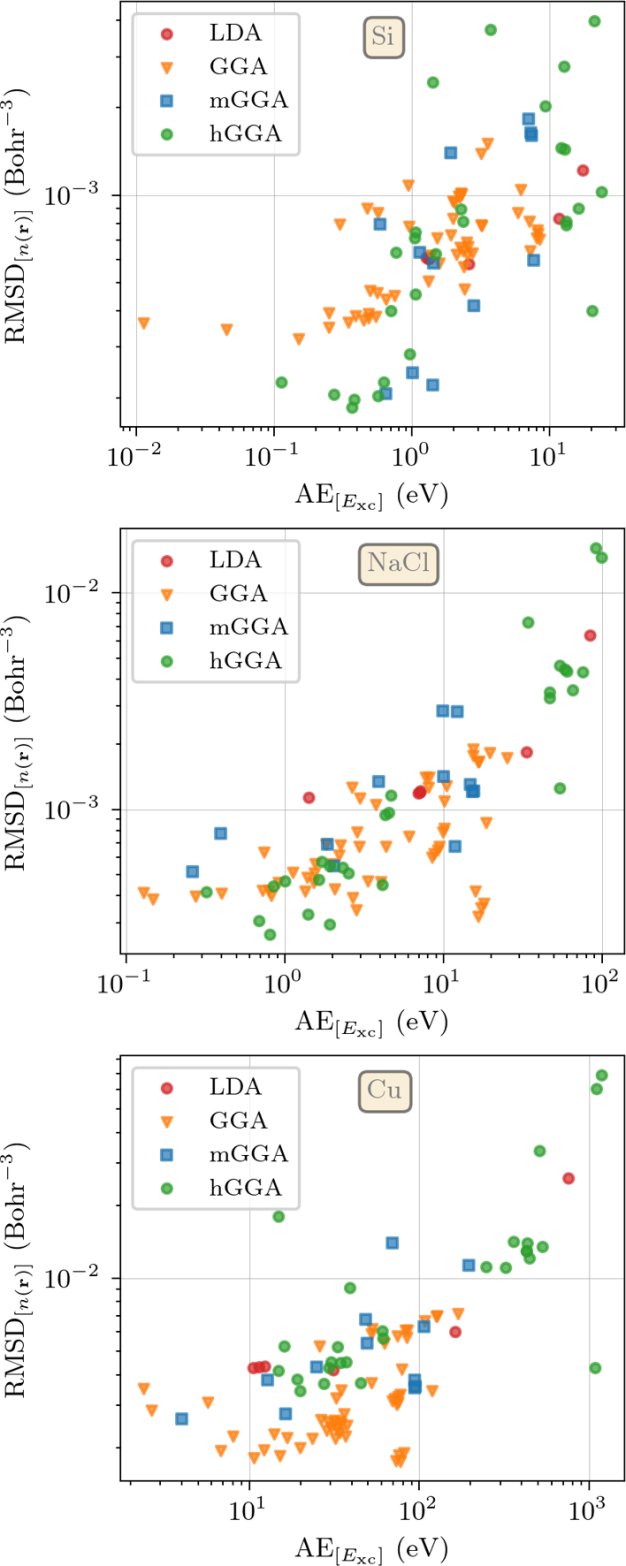
Distribution of RMSD_[*n*(**r**)]_ and AE_[*E*_xc_]_ for Si, NaCl, and Cu (top, middle, and bottom, respectively) as computed with all functionals considered in this work.

For the case of Si, Figure 1 depicts the normalized RMSD of the electron density RMSD_[*n*(**r**)]_ and of the exchange-correlation energy AE_[*E*_xc_]_ for each of the tested functionals. Overall, there does not appear to be a strong trend among all functionals, as corroborated by rank correlation analysis. For the complete set of 98 functionals, the Kendall’s τ value is 0.48. A similar analysis was conducted in terms of functional rungs, resulting in the following values: 0.33 for the LDAs (6 functionals), 0.45 for the GGAs (53 functionals), 0.36 for the mGGAs (12 functionals) and finally 0.56 for the hybrids (27 functionals).

Table 1 presents the 24 best performing functionals for the reproduction of the exact density of silicon, as ranked by their normalized RMSD_[*n*(**r**)]_. Of these, 15 were published before 2000. The top 10 functionals are hybrids or meta-GGAs, and with the exception of B3PW91, are known to generate accurate densities for the systems of ref (25). It is noteworthy that among these top-performing functionals, 17 are also among the top 24 functionals as ranked by the quality of the normalized AE_[*E*_xc_]_. A combined analysis of the performance of these functionals with respect to both quantities reveals that GGAs occupy a dominant position, ranking as the best DFT functionals for the xc energy. We remark that, despite yielding reasonable densities, M05 and MNL12-L are among the 25% least accurate functionals to evaluate xc energies.

**Table 1:** Top 24 Functionals Ranked by RMSD_[*n*(**r**)]_ for Si^a^

functional	rung	year	NRMSD	#RMSD_[*n*(**r**)]_	#AE_[*E*_xc_]_
mPW1PBE	hGGA	1998	0.279	1	10
APFD	hGGA	2012	0.298	2	11
PBE0	hGGA	1999	0.306	3	20
mPWPW91	hGGA	1998	0.310	4	7
SCAN	mGGA	2015	0.311	5	25
revTPSS	mGGA	2009	0.335	6	42
B3PW91	hGGA	1993	0.341	7	3
HSE06	hGGA	2006	0.342	8	23
TPSS	mGGA	2003	0.369	9	32
B3P86	hGGA	1993	0.426	10	31
GP86	GGA	1996	0.481	11	4
PW91P86	GGA	1991	0.518	12	2
GPW91	GGA	2001	0.528	13	6
BP86	GGA	1986	0.545	14	1
GPBE	GGA	1996	0.551	15	9
PW91	GGA	1992	0.558	16	13
mPWPBE	GGA	1998	0.568	17	15
PW91PBE	GGA	1997	0.573	18	18
BPW91	GGA	1991	0.578	19	12
BPBE	GGA	1996	0.589	20	16
PBEP86	GGA	1996	0.593	21	5
B97–2	hGGA	2001	0.601	22	26
M05	hGGA	2005	0.601	23	95
MN12-L	mGGA	2012	0.625	24	69

a For each functional we indicate the rung of the Jacob’s ladder, publication year, normalized RMSD_[*n*(**r**)]_ and their ranks according to RMSD_[*n*(**r**)]_ and AE_[*E*_xc_]_.

**Figure 2:**
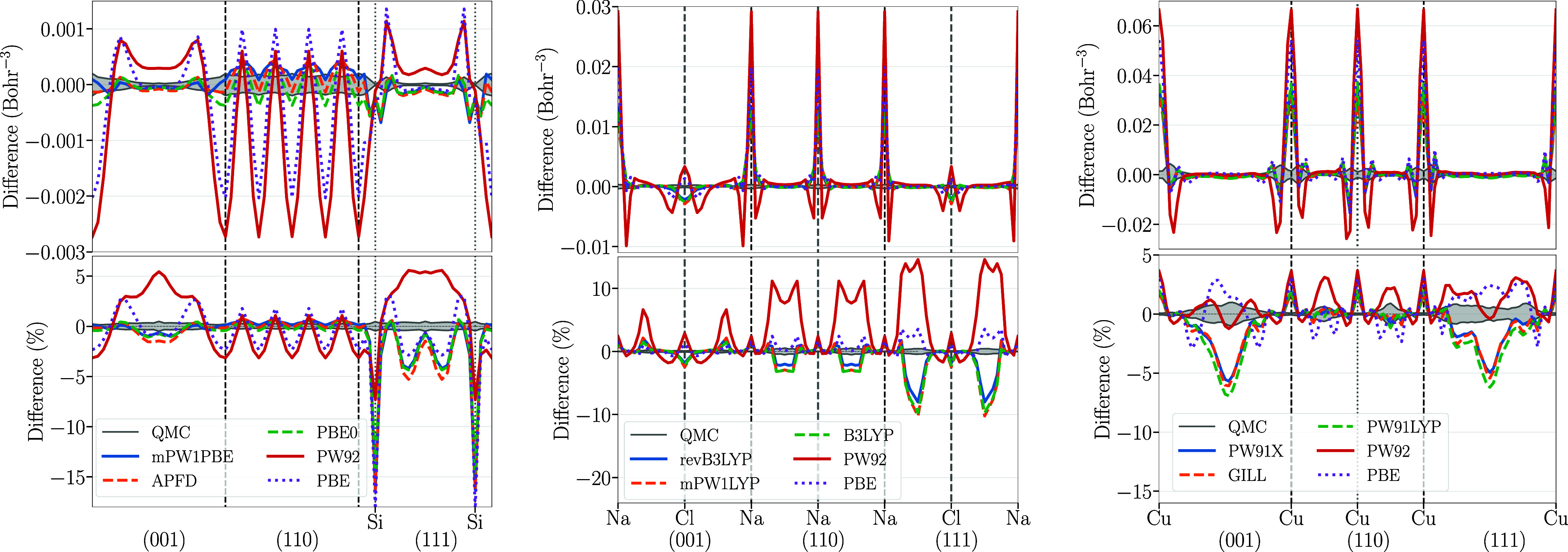
Difference (top panel) and relative difference (bottom panel) between the QMC densities and the DFT densities for Si, NaCl, and Cu. For each material, results for the three top performing functionals are plotted, together with PBE and PW92 calculations for reference. The gray area represents the expected error in the QMC density at the given point, while the dotted vertical lines represent atomic planes. For the exact definition of the high-symmetry lines in the unit cell, along the different crystallographic directions, we redirect the reader to Figure 4 of ref (26).

Since RMSD_[*n*(**r**)]_ is an integrated quantity, small values do not guarantee that the density is optimal at all points in space. Thus, to gain insight into the generated densities, we look at the difference between the DFT and QMC densities for the top three functionals of Table 1 (mPW1PBE, APFD and PBE0) within the unit cell. For clarity, both the difference and relative difference are plotted in Figure 2, along the same high-symmetry path in the unit cell that is described in Section 4 of ref (26). The path follows a triangular route *O* – < 001 > – *O*′– < 110 > – *O*″– < 111 > – *O*. The origin O is defined as the point midway between two adjacent Si atoms. Points *O*′ and *O*″ are obtained by translating *O* by one lattice constant along their respective connecting directions.

In general, all the functionals yield very small errors when compared to the uncertainty of the QMC data, however with a loss of accuracy along the low-density directions <111>. Both APFD and PBE0 outperform mPW1PBE in the <110> directions, where the density is close to its mean value and exhibits oscillations. Conversely, mPW1PBE appears to be more accurate in the <001> directions where the density is notably low. All functionals exhibit significant improvements when compared to the LDA (PW92) density, except for positions on top of the Si atoms.

**Figure 3:**
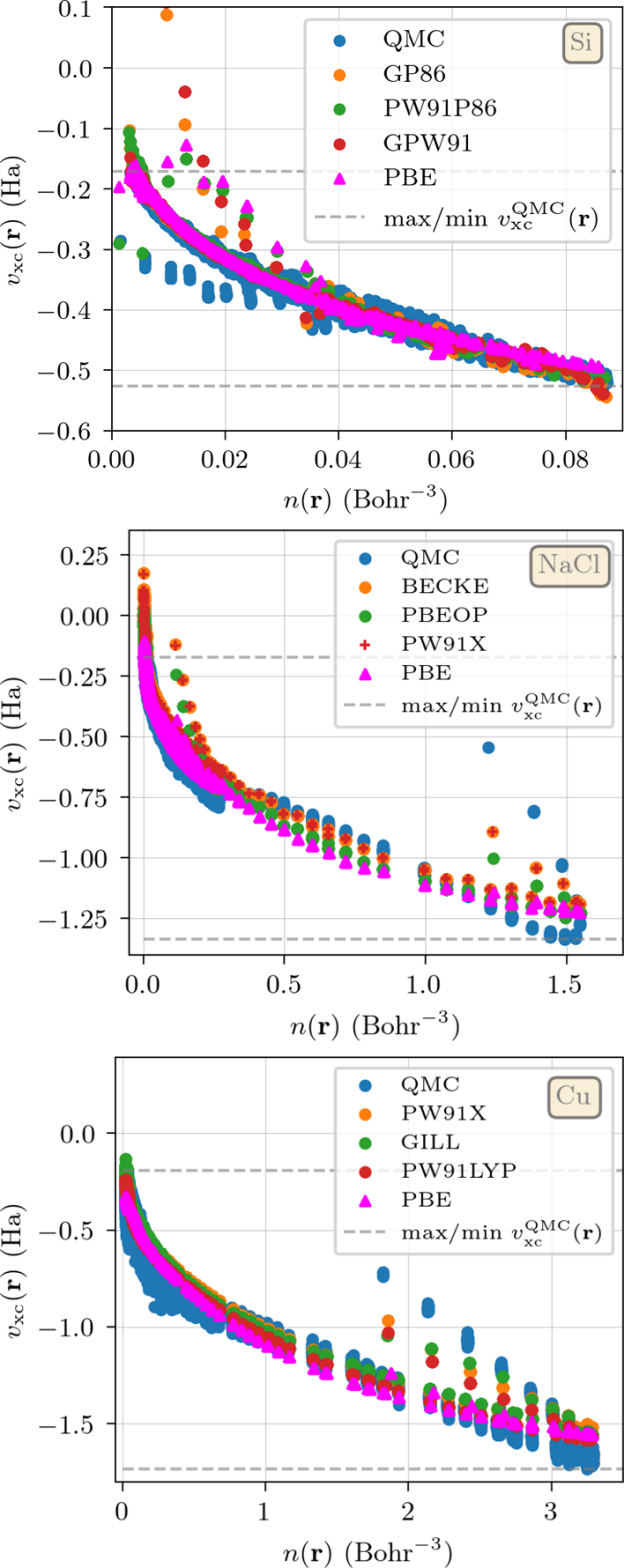
Parametric curve of (*n*(**r**), *v*_xc_[*n*](**r**)) for Si (top), NaCl (middle), and Cu (bottom), computed with the QMC data, as well using calculations obtained with PBE and the three top performing LDAs/GGAs for each material (see Tables 1–3). The values of the potential are computed with the corresponding ground-state density obtained from solving the Kohn–Sham equations. For the QMC reference, the potential was obtained by inversion of the Kohn–Sham equations. For better visualization, the upper (lower) dashed horizontal lines represent the maximum (minimum) value of *v*_xc_ obtained in the QMC calculations.

Another way to evaluate the quality of the different functionals is by looking at the xc potential *v*_xc_^DFT^(**r**). This map provides insight about the dependence of the potential on the local density, and in which regions nonlocal effects are important. It is therefore particularly meaningful for LDAs and GGAs (i.e., semilocal functionals). We remind that the reference value *v*_xc_^QMC^(**r**) is readily available by inverting the Kohn–Sham equations, following the procedure of ref (39). For Si we present the QMC results compared to the ones of GP86, PW91P86 and GPW91 (the top three GGAs of Table 1), as well as PBE, due to its popularity in the community. For the best performing functionals we can also observe the dependence of *v*_xc_[*n*](**r**) on the value of *n*(**r**) in Figure 3. The data points in Figure 3 can be divided in two branches. The main branch contains the highest density of points and is monotonically decreasing as the density increases. It has been shown in ref (39) that this branch is closer to the LDA *v*_xc_, due to its monotonic behavior as a function of *n*(**r**). The secondary branch lies below the main one in the region of densities satisfying *n*(**r**) ∈ [0, 0.04] and serves as an indicator of the nonlocal dependence of the xc potential on the density. The points in this branch can be characterized by a higher kinetic energy density and different density gradient than those on the main branch.

We can observe that the description of the main branch is overall good, independently on the considered functional, but the functionals tend to make a qualitatively wrong description of the secondary branch, predicting it to lie above the main one.

PBE is the most extreme case, since the entire secondary branch of *v*_xc_ lies above the main one. The two functionals incorporating the GILL exchange term exhibit a wider range of *v*_xc_ compared to the QMC data, with GP86 even showing positive values between the range of densities from 0.01 to 0.02 Bohr^–3^. They also predict some points in the correct side near *n*(**r**) ≈ 0.04 Bohr^–3^. As for PW91P86, in the low-density regime it somehow manages to both produce values of *v*_xc_ above the reference range and some correct points on the secondary branch.

This qualitative assessment suggests that PW91P86 is the most accurate GGA functional for describing the QMC xc potential of Si. For this material it also provides the second-best density among GGA functionals and the second-best xc energy of all the DFT functionals we have considered.

### 3.2 NaCl

We now turn our attention to NaCl which, being an ionic solid, presents more a localized charge density than Si.

As before, we first take a look at the RMSD_[*n*(**r**)]_ and RMSD_[*E*_xc_]_ distribution of NaCl. Computing Kendall’s τ for NaCl we obtain 0.58 when considering all functionals (98 in total), 1.00 for the LDAs (6 functionals), 0.44 for the GGAs (53 functionals), 0.27 for the meta-GGAs (12 functionals) and 0.70 for the hybrids (27 functionals). Overall this indicates a better correlation than in the case of Si, as it is also apparent from visual inspection of Figure 1, albeit the improvement is marginal.

In Table 2, we show the 24 best performing functionals for the density, ranked by their normalized RMSD_[*n*(**r**)]_, 16 of which are GGAs. Among these, 10 yield xc energies that rank in the top 25%, while 4 of the remaining are exchange-only functionals (and accordingly fail in providing accurate energies). As in the case of Si, several hybrids are also present in the top 24 for NaCl, but curiously only two of them (namely HSE06 and B3P86) are present in the top 24 for both materials. It is peculiar the absence of meta-GGAs in the list, proving that they have an inferior performance for this ionic compound.

**Table 2:** Top 24 Functionals Ranked by RMSD_[*n*(**r**)]_ for NaCl^a^

functional	family	year	NRMSD	#RMSD_[*n*(**r**)]_	#AE_[*E*_xc_]_
revB3LYP	hGGA	2013	0.385	1	12
mPW1LYP	hGGA	1998	0.431	2	30
B3LYP	hGGA	1993	0.445	3	8
BECKE	GGA	1988	0.466	4	78
BLYP35	hGGA	2009	0.476	5	20
PBEOP	GGA	1997	0.502	6	40
PW91X	GGA	1991	0.513	7	80
PBEX	GGA	1997	0.536	8	81
PBEPW91	GGA	1997	0.561	9	2
BLYP	GGA	1988	0.572	10	39
PBE	GGA	1996	0.581	11	4
PW91	GGA	1992	0.584	12	13
PW91PBE	GGA	1997	0.597	13	7
PBEP86	GGA	1996	0.601	14	1
B97–1	hGGA	1998	0.607	15	5
GILL	GGA	1996	0.612	16	76
MPWLYP1W	GGA	2005	0.612	17	18
PBELYP1W	GGA	2005	0.614	18	9
PW91P86	GGA	1991	0.615	19	11
GLYP	GGA	1996	0.623	20	33
HSE06	hGGA	2006	0.643	21	14
B3P86	hGGA	1993	0.653	22	48
mPWPBE	GGA	1998	0.667	23	15
BP86	GGA	1986	0.671	24	22

a For each functional we indicate the rung of the Jacob’s ladder, publication year, normalized RMSD_[*n*(**r**)]_ and their ranks according to RMSD_[*n*(**r**)]_ and AE_[*E*_xc_]_.

As before, it is instructive to take a look at the charge density as well. In Figure 2, we present the comparison of the QMC density and the densities computed with revB3LYP, mPW1LYP, and B3LYP. There are only minor differences to be noted among the three functionals. Qualitatively, they all predict an excess (depletion) of charge density at the Na (Cl) atoms with respect to the QMC results. The interstitial regions along the <111> direction, where the density is particularly low, also present a depletion of charge with respect to the QMC reference. Quantitatively, revB3LYP takes a slight lead in reproducing the QMC density, albeit by a very small margin.

We can now compare the (*n*(**r**), *v*_xc_[*n*](**r**)) curves for NaCl in Figure 3. The plot shows QMC results together with calculations performed with the top three GGA functionals in Table 2: BECKE, PBEOP and PW91X, as well as PBE for comparison. Contrary to Si, here we can divide the curve into three branches.^39^ The first branch contains most of the points, and is located between *n*(**r**) values of 0 and 0.4 Bohr^–3^. The second branch starts above the first one and extends from 0.4 up to approximately *n*(**r**) ≈ 1.5 Bohr^–3^. Finally the third branch appears at higher densities and shows lower values of |*v*_xc_|. The exchange-only potentials, BECKE and PW91X, seem to perform very similarly, outperforming PBE in reproducing the middle branch up to *n*(**r**) ≈ 1 Bohr^–3^. Furthermore, they provide a more accurate description of the third branch at high densities, which corresponds to the region around the Na atoms and is characterized by rapidly varying density gradients. The PBEOP xc potential, whose RMSD_[*n*(**r**)]_ is lower than that of PBE, displays a more pronounced branch in the high-density region compared to PBE. This suggests that the relatively lower quality of the PBE density is due to its correlation part. Among the plotted functionals, only PBE yields an accurate xc energy, ranking in the fourth position for NaCl.

### 3.3 Cu

We consider now Cu as a prototypical metal. The rank correlation analysis between the RMSD_[*n*(**r**)]_ and AE_[*E*_xc_]_ for Cu returns a value for Kendall’s τ of 0.46 for all functionals (98 in total), 0.73 for the LDAs (6 functionals), 0.44 for the GGAs (53 functionals), 0.27 for the meta-GGAs (12 functionals) and 0.58 for the hybrids (27 functionals). Overall this indicates that no strong correlation is found, with the values showing a qualitative situation similar to the case of Si.

In Table 3, we present the 24 best performing functionals for the reproduction of the exact density for Cu, as ranked by their normalized RMSD_[*n*(**r**)]_. As can be seen, the entire list is dominated by GGAs. The absence of (global, unscreened) hybrids is expected, due to the well-known problem of Hartree–Fock calculations that lead to a nonphysical, vanishing density of states at the Fermi level. One peculiar thing about this list is that the exchange-only functionals tend to perform quite well when it comes to reproduce the charge density, with PW91X, GILL, BECKE and PBEX taking first, second, fifth, and sixth places on the list (showing an unsurprising poor performance for the prediction of the exchange-correlation energy). Adding LYP correlation to PW91 and PBE results in an overall improvement in performance, as PW91LYP (PBELYP) keeps a good description of density at third place (seventh) in terms of RMSD_[*n*(**r**)]_, but rises to eighth (fifth) in terms of AE_[*E*_xc_]_, up from the 69th (71st) of its exchange-only counterpart. GILL also yields a similar, but smaller, rise in performance (with GLYP being 16th in terms of AE_[*E*_xc_]_). When taking both RMSD_[*n*(**r**)]_ and AE_[*E*_xc_]_ into consideration, LYP correlation seems to outperform other approximations to the correlation potential when added to exchange-only functionals. This is clear when comparing the results of PW91LYP against those of PW91P86 and PW91PBE, BLYP against BOP and BP86, GLYP against GOP, GP86, GPW91 and GPBE, and PBELYP against PBEOP, PBEP86 and PBE.

**Table 3:** Top 24 Functionals Ranked by RMSD_[*n*(**r**)]_ for Cu^a^

functional	rung	year	NRMSD	#RMSD_[*n*(**r**)]_	#AE_[*E*_xc_]_
PW91X	GGA	1991	0.459	1	69
GILL	GGA	1996	0.463	2	62
PW91LYP	GGA	1991	0.475	3	8
GLYP	GGA	1996	0.486	4	16
BECKE	GGA	1988	0.490	5	67
PBEX	GGA	1997	0.499	6	71
PBELYP	GGA	1997	0.510	7	5
BLYP	GGA	1988	0.511	8	10
MPWLYP1W	GGA	2005	0.522	9	21
PBELYP1W	GGA	2005	0.571	10	23
PW91P86	GGA	1991	0.572	11	36
GOP	GGA	2001	0.576	12	19
GP86	GGA	1996	0.584	13	46
PBEOP	GGA	1997	0.585	14	6
BOP	GGA	2001	0.596	15	13
BP86	GGA	1986	0.606	16	40
PBEP86	GGA	1996	0.616	17	28
PW91	GGA	1992	0.625	18	38
PW91PBE	GGA	1997	0.636	19	31
GPW91	GGA	2001	0.649	20	48
mPWPBE	GGA	1998	0.655	21	34
GPBE	GGA	1996	0.658	22	43
BPW91	GGA	1991	0.669	23	42
PBEPW91	GGA	1997	0.672	24	29

a For each functional we indicate the rung of the Jacob’s ladder, publication year, normalized RMSD_[*n*(**r**)]_ and their ranks according to RMSD_[*n*(**r**)]_ and AE_[*E*_xc_]_.

For the sake of simplicity, from here on we will focus simply on PW91X, GILL and PW91LYP. In spite of their overall performance, when looking at the local description of the density (see Figure 2) we observe that all three functionals have problems in describing the interstitial low-density regions, presenting a general underestimation. This is not unexpected, as it follows the general trend observed in Si and NaCl and curiously, both PW92 and PBE seem to perform better here at the expense of a poorer description of the near-nuclei regions.

Turning to the (*n*(**r**), *v*_xc_[*n*](**r**)) map of Cu, as shown in Figure 3, we see that this curve seems qualitatively similar to that of NaCl. Concerning the main branch, we can distinguish two regions: up to 0.5 Bohr^–3^ a steep variation in *v*_xc_[*n*] is observed after which the curve becomes shallower. For densities above 2 Bohr^–3^ a second branch above the main one can be seen, which meets back with the main one slightly above 3 Bohr^–3^. The maps for PW91X, GILL, PW91LYP and PBE present a qualitatively similar behavior, being always slightly “off” with respect to the exact data. Within the main branch, at low and high densities these functionals tend to overestimate *v*_xc_, while at intermediate values the opposite is true, leading to a notorious bowing effect; within the secondary branch, all functionals underestimate *v*_xc_. GILL and PW91X (exchange-only functionals) are quantitatively very close, albeit GILL does predict values of *v*_xc_ above the reference data for low densities. The inclusion of correlation in PW91LYP seems to worsen the description of both the intermediate density regions of the main branch and the secondary branch, associated with a further reduction of *v*_xc_ in those areas. This change is not associated with an improvement of the description of the regions where PW91X overestimates the reference data. As a comparison, PBE shows the worst performance among these functionals, presenting a very pronounced bowing and underestimating *v*_xc_ in the secondary branch to the point of almost placing this branch atop the main one.

### 3.4 General Observations

Looking at Tables 1–3 concerning the best results for RMSD_[*n*(**r**)]_, we see that GGA is the most represented family of functionals. Among these, combinations of either component of BP86, PBE, PW91 are the most common. PBEP86 stands out as being the only functional simultaneously present on the three top 24 lists, surprisingly returning consistent values of normalized RMSD_[*n*(**r**)]_ of 0.6. This functional comes from two different publications, being an admixture of PBE exchange and P86 correlation. With the exception of exchange-only GGAs, most of these functionals also appear in the top 24 as ranked by AE_[*E*_xc_]_ (as computed for all considered crystalline materials), indicating good performance in both fronts, and weighting in favor of focusing on exact constraints for the construction of functionals. In spite of the very different nature of NaCl and Cu, a good description of both their densities can be achieved with exchange-only functionals with PW91X, BECKE, PBEX and GILL appearing in the leaderboards.

On another rung, hybrid functionals show good performance for Si and NaCl. Of the top performing hybrids for these materials only two are simultaneously on both leaderboards: HSE06 and B3P86. Their performance varies somewhat between the two materials: HSE06 stands as the eighth (23rd) best functional in terms of RMSD_[*n*(**r**)]_ (AE_[*E*_xc_]_) for Si, while it is the 21st (14th) best in the same terms for NaCl; B3P86 takes the 10th (31st) place in terms of RMSD_[*n*(**r**)]_ (AE_[*E*_xc_]_) for Si, while it is the 22nd (48th) best in the same terms for NaCl. Despite the different relative ranking of these functionals for the two materials, the associated absolute errors are similar, and both functionals have advantage of performing very well for the set of systems studied in ref (25), which represents a very different chemical environment. It is curious to notice that these functionals are considerably different in their construction.

With regard to meta-GGAs, they are only present in the top24 of Si, where we find well-known names such as SCAN, TPSS and revTPSS.

We finally remark that, in spite of their integrated performance, the top performing functionals for all materials tend to struggle in the description of low-density interstitial regions.

As a point of comparison, in the literature several benchmarks on the density exist for atoms (in particular closed-shell ones) and molecules (e.g.,^37,41−45^). The reference data is in that case typically obtained using post-HF methods (such as CCSDTQ) and large basis sets. For those systems, the performance of meta-GGAs is clearly better than the performance of GGAs and slightly better or comparable to the one of hybrid functionals. Particularly good are SCAN and TPSS, which rank among the top functionals in our results for Si (although not for NaCl and Cu). Overall, GGAs tend to perform worse for molecules and atoms than in for solids. This fact could be to a certain extent expected, as solids are less inhomogeneous than finite systems. It is however unclear why meta-GGAs and hybrid functionals perform significantly worse for NaCl and Cu than for Si.

### 3.5 Historical Trends

As discussed in the introduction, one of the critics in modern functional construction concerns the exaggerated fitting, which leads to good energy values but poor density description. The question arises whether this is the case for the present systems, similarly to what was observed in ref (25).

**Figure 4:**
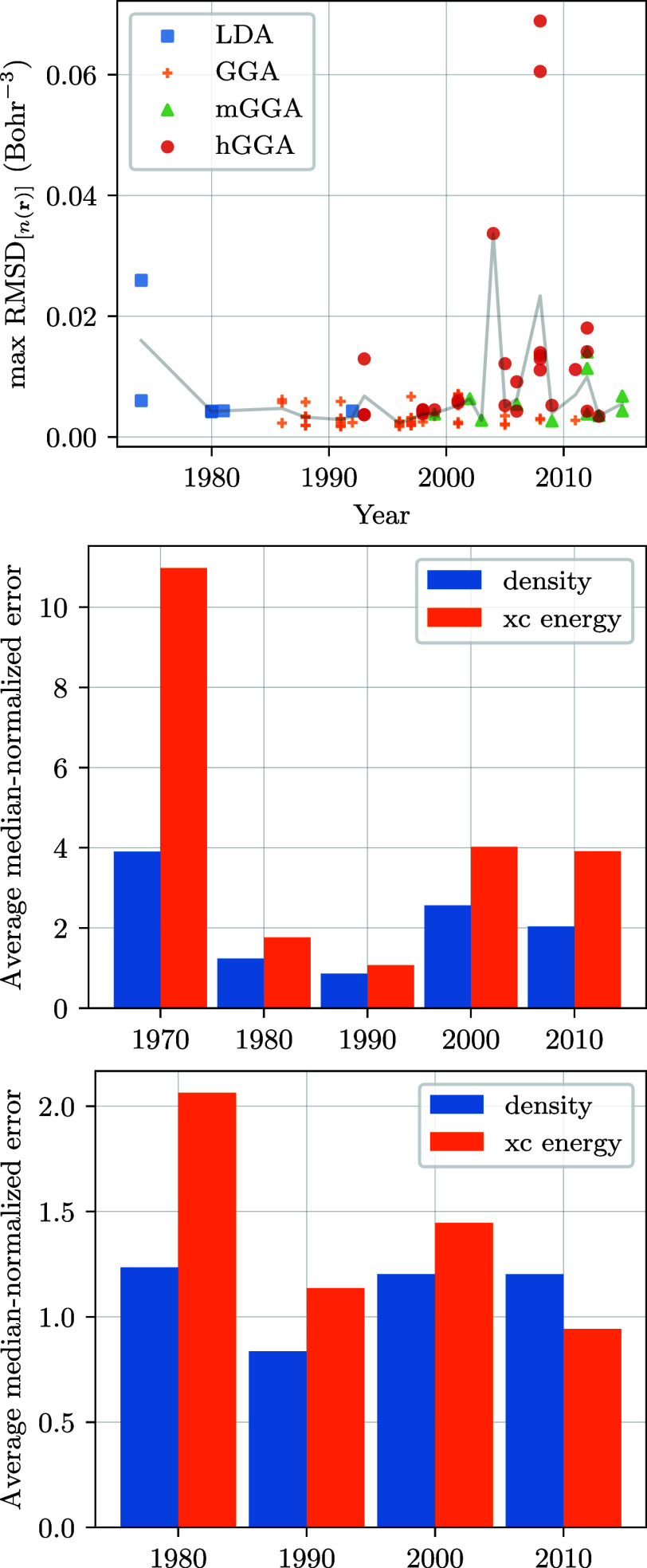
Analysis of the historical evolution of errors for the xc functionals and systems under study. The first image (top) shows the maximal RMSD of the density resulting from DFT functionals in function of their publication years. The line shows the mean of RMSD for each year. The second image (middle) presents the historical trends of the DFT functionals performance on the xc energy and the density. The histogram shows the maximal normalized-RMSD and normalized-AE per publication decade for density and energy, respectively. The last image (bottom) shows the same as before but for the GGA family only.

First, from the results of Tables 1–3, we see that the best functionals for the description of the density come from before 2000. Figure 4a plots the evolution of average RMSD_[*n*(**r**)]_ as a function of publication year, showing on average an improvement in quality up to the mid-1990s, after which a notable fluctuation in the error is observed (in line with the findings of ref (25)). The most “egregious” culprits of this behavior are hybrid functionals such as wB97 (2008), CAMB3LYP (2004), and MN12SX (2012), all of which have indeed been fitted to different types of thermo-chemical data.

Looking alone at the evolution of the RMSD_[*n*(**r**)]_, however, does not provide the complete historical picture. In Figure 4b, we thus show the average of RMSD_[*n*(**r**)]_ and AE_[*E*_xc_]_ for each decade of the functionals under consideration. For the extended systems we have considered, and similarly to the density case, the results indicate that the AE_[*E*_xc_]_ improve until the end of the previous century. However, for more modern DFT functionals, we observe a proportional decrease in the quality of the reproduction of the xc energy and the quality of the density at the same time. If we consider only GGAs (Figure 4c), this trend is kept until the early 2000s, at which point the error in the energy increases and then slightly decreases again in 2010. In addition, the average error in the energy improves significantly with time, with errors on the density being only marginally affected.

## 4 Conclusions

In conclusion, we leveraged recent nearly exact calculations of electron densities for silicon, sodium chloride, and copper to benchmark 98 approximate xc functionals from most rungs of Jacob’s Ladder. This comprehensive analysis revealed no large-scale correlation between errors in charge density and xc energy, consistent with previous literature observations.

Interestingly, even for these three relatively simple materials, some heavily fitted functionals like M05 and MN12-L fail to provide both accurate densities and xc energies, raising questions about transferability of such functionals. In contrast, a handful of GGAs offer decent descriptions of both xc energies and densities, likely due to their use of exact constraints, which appears to enhance transferability across different materials.

For the first time, we inverted the xc potential of copper and qualitatively analyzed its (*n*(**r**), *v*_xc_[*n*](**r**)) map, providing new insights into metallic systems. Our findings indicate that for this metal, LYP approximation of the correlation energy seems to outperform other approximations.

The hybrid functional HSE06 shows good performance for both energy and densities in the ionic solid and the semiconductor, but not for the metal. Interestingly, the PBE correlation part seems responsible for the poor description of xc potential in high-density regions of sodium chloride and copper. Using OP or LYP correlation instead can improve the reproduction of density and xc potential in these materials.

Historical trends reveal a proportional decrease in the quality of xc energy reproduction and density accuracy. Both metrics improved up to around 2000, after which they deteriorated for the solids studied. While this may support arguments about overfitting in modern functionals, it also reflects evolving research priorities, such as the focus on accurate band gap calculations at the expense of density and xc energy accuracy.

The availability of nearly exact data for more materials would be invaluable for further improving functionals for solids. We hope this work motivates the community to explore this direction, emphasizing the importance of balancing accuracy in both density and energy calculations for developing more reliable and transferable functionals.

## Supporting Information Available

Abbreviations for the considered rungs of exchange-correlation functionals; list of considered exchange-correlation functionals; error metric; convergence error; and top 24 functionals ranked by NIAD[n(r)] for Si, NaCl, and Cu (PDF)
