# Supplementary material for: Assessing Exchange-Correlation Functionals for Accurate Densities of Solids
Source: J Chem Theory Comput. 2024 Dec 3;20(24):10852–60. doi: 10.1021/acs.jctc.4c01042 (PMC11672669; doi:10.1021/acs.jctc.4c01042)
Supplement: Supplementary file 1 — ct4c01042_si_001.pdf [file ct4c01042_si_001.pdf]

Supporting information: Assessing exchange-correlation functionals for accurate densities of solids

Ayoub Aouina,[1, 2, 3] Pedro Borlido,[4] Miguel A.L. Marques,[1, 3] and Silvana Botti[1, 2, 3]

[1]*Research Center Future Energy Materials and Systems of the University Alliance Ruhr and Interdisciplinary Centre for Advanced Materials Simulation, Ruhr University Bochum, Universitätsstraße 150, D-44801 Bochum, Germany*
[2]*Institut für Festkörpertheorie und -optik, Friedrich-Schiller-Universität Jena, D-07743 Jena, Germany*
[3]*European Theoretical Spectroscopy Facility (ETSF)*[*]
[4]*CFisUC, Department of Physics, University of Coimbra, Rua Larga, 3004-516 Coimbra, Portugal*

S1. ABBREVIATIONS FOR THE CONSIDERED RUNGS OF EXCHANGE-CORRELATION FUNCTIONALS

- LDA local density approximation
- GGA generalized gradient approximation
- mGGA meta generalized gradient approximation
- hGGA hybrid functionals with GGA correlation

S2. LIST OF CONSIDERED EXCHANGE-CORRELATION FUNCTIONALS

Here we indicate the complete list of exchange-correlation functionals considered for the Kohn-Sham calculations in this work. Some of the functionals were also used in Ref. [1].

- LDA functionals: SLATER [2, 3], SVWN [4], SVWN5RPA [4], SPZ81 [5] , Xα [6], SPW92 [7];
- GGAs: BP86 (BECKE exchange [8] + P86 correlation [9]), SP86 (SLATER exchange + P86 correlation), OP86 (OPTX exchange [10] + P86 correlation), BECKE (exchange only) [8], SLYP (SLATER exchange + LYP correlation [11, 12]), BPZ81 (BECKE exchange + PZ81 correlation), BVWN5RPA (BECKE exchange + SVWN5RPA correlation), BVWN (BECKE exchange + SVWN correlation), PW91X (exchange only) [13, 14], PW91VWN (PW91 exchange + SVWN correlation), PW91PZ81 (PW91 exchange + PZ81 correlation), PW91P86 (PW91 exchange + P86 correlation), PW91LYP (PW91 exchange + LYP correlation), SPW91 (SLATER exchange + PW91 correlation), BPW91 (BECKE exchange + PW91 correlation), PW91, PBEP86 (PBE exchange + P86 correlation), GPBE (GILL exchange [15] + PBE correlation), PBE, BPBE (BECKE exchange + PBE correlation), GP86 (GILL exchange + P86 correlation), GLYP (GILL exchange + LYP correlation) , GILL (exchange only), PBEPW91 (PBE exchange + PW91 correlation), PBEOP (PBE exchange + OP correlation [16, 17]), PBELYP (PBE exchange + LYP correlation), PBEX (exchange only), PW91PBE (PW91 exchange + PBE correlation) , HCTH407 [18], PBEPZ81 (PBE exchange + PZ81 correlation), PBEVWN (PBE exchange + VWN correlation), EDF1 [19], revPBE [20], mPWPBE (mPW exchange [21] + PBE correlation), RPBE (RPBE exchange [22] + PBE correlation), BOP (BECKE exchange + OP correlation), GPW91 (GILL exchange + PW91 correlation), OVWN (OPTX exchange + VWN correlation), OVWN5RPA (OPTX exchange + VWN5RPA correlation) , GOP (GILL exchange + OP correlation), OPW91 (OPTX exchange + PW91 correlation), OPTX (exchange only), OPBE (OPTX exchange + PBE correlation) , OLYP (OPTX exchange + LYP correlation) , OPZ81 (OPTX exchange + PZ81 correlation) , PBELYP1W [23] , MPWLYP1W [23], MOHLYP [24], BLYP (BECKE exchange + LYP correlation), SOGGA (SOGGA exchange [25] + PBE correlation), PBEsol [26], SOGGA11 [27], N12 [28];
- mGGAs: PKZB [29], TPSS [30, 31], M06-L [32, 33], revTPSS [34, 35], M11-L [36], MN12-L [37], MS0 (exchange only) [38], MS1 [39], MS2 [39], MVS [40], SCAN [41];

---

[*] ayoub.aouina@rub.de

	Mean Conv. Error	Max Conv. Error	Min AE$_{[E_{xc}]}$	Max AE$_{[E_{xc}]}$	Mean AE$_{[E_{xc}]}$	5% Percentile AE$_{[E_{xc}]}$
Si	0.484	2.232	0.011	23.713	3.821	0.249
NaCl	0.344	0.527	0.129	99.550	13.454	0.385
Cu	1.726	2.332	2.391	1179.101	122.782	7.836

TABLE SI. Convergence error for ill-behaved functionals and AE$_{[E_{xc}]}$ statistics in eV for Si, Cu, and NaCl.

- hGGAs: B3LYP [42], B3P86 [43], B3PW91 [43], mPWPW91 [21], B97-1 [44], mPW1LYP [21], mPW1PBE [21], PBE0 [45, 46], B97-2 [47], O3LYP [48, 49], CAM-B3LYP [50], B97-3 [51], M05 [52], HSE06 [53–55], M06-HF [56], M08-HX [57], M08-SO [57], M06 [33], M06-2X [33], BLYP35 [58, 59], M11 [60], APFD [61], MN12-SX [62], N12-SX [62], revB3LYP [63].

S3. ERROR METRIC

It should be noted that RMSD$_{[n(\mathbf{r})]}$ as defined in the main text is not size consistent and, being a square root measure, gives larger errors a disproportionately large effect [64]. One metric that has been proposed in the literature to cure this problem is the normalized integral absolute deviation (NIAD)

$$\mathrm{NIAD}_{[n(\mathbf{r})]} = \frac{1}{N_e}\int \mathrm{d}\mathbf{r}\,|n^{\mathrm{DFT}}(\mathbf{r}) - n^{\mathrm{QMC}}(\mathbf{r})| \tag{S1}$$

where N_e is the number of electrons. Similarly to the tables presented in the main text, Table SII, SIII, and SIV show the top 24 functionals ranked by NIAD$_{[n(\mathbf{r})]}$. For Si, the NIAD and RMSD metrics yield almost the same functionals, except for PBE, which is ranked 26th by RMSD and 23rd by NIAD. In the case of NaCl, NIAD includes BPBE and BPW91 in the list over HSE06 and GILL, which are ranked 25th and 26th by NIAD, respectively. For Cu, the only new-appearing functional is PBEsol, which replace GPBE. It is interesting to note how closely the two metrics agree on the top-performing functionals for the three materials, even though their positions differ slightly. This suggests that the ranking is globally robust.

S4. CONVERGENCE ERROR

In the main text, we mention that some meta-GGA functionals show slower convergence and their convergence errors on the total energy are larger compared to other functionals. The complete list of these functionals for each materials is as follows:

- For Si: MN12L, M11L, M06L, SCAN, MS0, MS1, MVS.
- For NaCl: MN12L, M11L, TPSS, M06L, PKZB, revTPSS, and SCAN.
- For Cu: MN12L, M11L, M06L, and MVS.

It is important to note that the convergence error of these functionals is not always a significant issue, as in many cases the AE$_{[E_{xc}]}$ is much larger. In Table S4, for each material, we present the mean and maximum convergence errors for these ill-behaved functionals, alongside the minimum, maximum, mean, and 5% percentile of AE$_{[E_{xc}]}$ of all functionals.

[1] M. G. Medvedev, I. S. Bushmarinov, J. Sun, J. P. Perdew, and K. A. Lyssenko, Science **355**, 49 (2017).
[2] F. Bloch, Zeitschrift für Physik **57**, 545 (1929).
[3] P. A. M. Dirac, Mathematical Proceedings of the Cambridge Philosophical Society **26**, 376 (1930).
[4] S. H. Vosko, L. Wilk, and M. Nusair, Canadian Journal of Physics **58**, 1200 (1980).
[5] J. P. Perdew and A. Zunger, Physical Review B **23**, 5048 (1981).
[6] Á. Nagy, International Journal of Quantum Chemistry **31**, 269 (1987).
[7] J. P. Perdew and Y. Wang, Physical Review B **45**, 13244 (1992).
[8] A. D. Becke, The Journal of Chemical Physics **84**, 4524 (1986).
[9] J. P. Perdew, Physical Review B **33**, 8822 (1986).

Functional	Rung	Year	NRMSD	NIAD	#RMSD$_{[n(\mathbf{r})]}$	#NIAD$_{[n(\mathbf{r})]}$	AE$_{[E_{\mathrm{xc}}]}$
mPW1PBE	hGGA	1998	0.2797	0.0381	1	1	10
APFD	hGGA	2012	0.2981	0.0428	2	2	11
SCAN	mGGA	2015	0.3119	0.0432	5	3	25
PBE0	hGGA	1999	0.3060	0.0435	3	4	20
mPWPW91	hGGA	1998	0.3105	0.0442	4	5	7
revTPSS	mGGA	2009	0.3350	0.0473	6	6	42
B3PW91	hGGA	1993	0.3415	0.0500	7	7	3
TPSS	mGGA	2003	0.3692	0.0502	9	8	32
HSE06	hGGA	2006	0.3421	0.0504	8	9	23
B3P86	hGGA	1993	0.4263	0.0618	10	10	31
GP86	GGA	1996	0.4812	0.0622	11	11	4
GPW91	GGA	2001	0.5282	0.0628	13	12	6
GPBE	GGA	1996	0.5512	0.0634	15	13	9
M05	hGGA	2005	0.6014	0.0648	23	14	96
PW91PBE	GGA	1997	0.5740	0.0684	18	15	18
mPWPBE	GGA	1998	0.5682	0.0692	17	16	15
PW91	GGA	1992	0.5585	0.0697	16	17	13
PW91P86	GGA	1991	0.5190	0.0700	12	18	2
BPBE	GGA	1996	0.5899	0.0717	20	19	16
BPW91	GGA	1991	0.5790	0.0735	19	20	12
BP86	GGA	1986	0.5456	0.0742	14	21	1
PBEP86	GGA	1996	0.5932	0.0783	21	22	5
PBE	GGA	1996	0.6798	0.0800	26	23	27
PBEPW91	GGA	1997	0.6585	0.0808	25	24	24

TABLE SII. Top 24 functionals ranked by NIAD$_{[n(\mathbf{r})]}$ for Si. For each functional we indicate the rung of the Jacob's ladder, publication year, normalized RMSD$_{[n(\mathbf{r})]}$, NIAD and their ranks according to the different metrics considered.

Functional	Rung	Year	NRMSD	NIAD	#RMSD$_{[n(\mathbf{r})]}$	#NIAD$_{[n(\mathbf{r})]}$	#AE$_{[E_{\mathrm{xc}}]}$
revB3LYP	hGGA	2013	0.3845	0.0297	1	1	12
BECKE	GGA	1988	0.4653	0.0328	4	2	79
PW91	GGA	1992	0.5827	0.0384	12	3	13
B3LYP	hGGA	1993	0.4440	0.0392	3	4	8
GLYP	GGA	1996	0.6221	0.0392	20	5	33
PW91X	GGA	1991	0.5124	0.0401	7	6	81
PW91PBE	GGA	1997	0.5956	0.0405	13	7	7
mPW1LYP	hGGA	1998	0.4301	0.0409	2	8	30
PBEPW91	GGA	1997	0.5603	0.0421	9	9	2
PBEX	GGA	1997	0.5345	0.0439	8	11	82
BP86	GGA	1986	0.6697	0.0439	24	10	22
PBE	GGA	1996	0.5794	0.0440	11	12	4
PBEOP	GGA	1997	0.5014	0.0441	6	13	40
BLYP	GGA	1988	0.5705	0.0459	10	14	39
mPWPBE	GGA	1998	0.6659	0.0475	23	15	15
MPWLYP1W	GGA	2005	0.6112	0.0519	17	16	18
PW91P86	GGA	1991	0.6136	0.0542	19	17	11
BLYP35	hGGA	2009	0.4754	0.0543	5	18	20
PBEP86	GGA	1996	0.5999	0.0550	14	19	1
BPW91	GGA	1991	0.7368	0.0586	30	20	23
B3P86	hGGA	1993	0.6516	0.0604	22	21	48
BPBE	GGA	1996	0.7451	0.0612	32	22	17
B97_1	hGGA	1998	0.6058	0.0614	15	23	5
PBELYP1W	GGA	2005	0.6127	0.0618	18	24	9

TABLE SIII. Top 24 functionals ranked by NIAD$_{[n(\mathbf{r})]}$ for NaCl. For each functional we indicate the rung of the Jacob's ladder, publication year, normalized RMSD$_{[n(\mathbf{r})]}$, NIAD and their ranks according to the different metrics considered.

Functional	Rung	Year	NRMSD	NIAD	#RMSD$_{[n(\mathbf{r})]}$	#NIAD$_{[n(\mathbf{r})]}$	#AE$_{[E_{\mathrm{xc}}]}$
PW91X	GGA	1991	0.4590	0.2371	1	1	69
PBEX	GGA	1997	0.4990	0.2511	6	2	71
GILL	GGA	1996	0.4633	0.2559	2	3	62
PW91LYP	GGA	1991	0.4754	0.2626	3	4	8
BECKE	GGA	1988	0.4904	0.2635	5	5	67
PBELYP	GGA	1997	0.5103	0.2736	7	6	5
GLYP	GGA	1996	0.4868	0.2810	4	7	16
PW91P86	GGA	1991	0.5723	0.2859	11	8	36
BLYP	GGA	1988	0.5114	0.2908	8	9	10
MPWLYP1W	GGA	2005	0.5222	0.3066	9	10	21
GP86	GGA	1996	0.5846	0.3156	13	11	46
BP86	GGA	1986	0.6063	0.3179	16	12	40
PBEP86	GGA	1996	0.6165	0.3211	17	13	28
PBEOP	GGA	1997	0.5850	0.3292	14	14	6
PBELYP1W	GGA	2005	0.5707	0.3338	10	15	23
GOP	GGA	2001	0.5762	0.3430	12	16	19
PW91	GGA	1992	0.6254	0.3473	18	17	38
BOP	GGA	2001	0.5963	0.3502	15	18	13
PW91PBE	GGA	1997	0.6361	0.3604	19	19	31
mPWPBE	GGA	1998	0.6555	0.3777	21	20	34
GPW91	GGA	2001	0.6492	0.3803	20	21	48
PBEsol	GGA	2008	0.7464	0.3838	30	22	2
BPW91	GGA	1991	0.6695	0.3853	23	23	42
PBEPW91	GGA	1997	0.6718	0.3909	24	24	29

TABLE SIV. Top 24 functionals ranked by NIAD$_{[n(\mathbf{r})]}$ for Cu. For each functional we indicate the rung of the Jacob's ladder, publication year, normalized RMSD$_{[n(\mathbf{r})]}$, NIAD and their ranks according to the different metrics considered.

[10] N. C. HANDY and A. J. COHEN, Molecular Physics **99**, 403 (2001).
[11] C. Lee, W. Yang, and R. G. Parr, Physical Review B **37**, 785 (1988).
[12] B. Miehlich, A. Savin, H. Stoll, and H. Preuss, Chemical Physics Letters **157**, 200 (1989).
[13] J. P. Perdew, J. A. Chevary, S. H. Vosko, K. A. Jackson, M. R. Pederson, D. J. Singh, and C. Fiolhais, Physical Review B **46**, 6671 (1992).
[14] J. P. Perdew, J. A. Chevary, S. H. Vosko, K. A. Jackson, M. R. Pederson, D. J. Singh, and C. Fiolhais, Physical Review B **48**, 4978 (1993).
[15] P. M. W. GILL, Molecular Physics **89**, 433 (1996).
[16] T. Tsuneda, T. Suzumura, and K. Hirao, The Journal of Chemical Physics **110**, 10664 (1999), https://pubs.aip.org/aip/jcp/article-pdf/110/22/10664/10798297/10664_1_online.pdf.
[17] T. Tsuneda, T. Suzumura, and K. Hirao, The Journal of Chemical Physics **111**, 5656 (1999), https://pubs.aip.org/aip/jcp/article-pdf/111/13/5656/10801160/5656_1_online.pdf.
[18] A. D. Boese and N. C. Handy, The Journal of Chemical Physics **114**, 5497 (2001), https://pubs.aip.org/aip/jcp/article-pdf/114/13/5497/10831004/5497_1_online.pdf.
[19] R. D. Adamson, P. M. Gill, and J. A. Pople, Chemical Physics Letters **284**, 6 (1998).
[20] Y. Zhang and W. Yang, Phys. Rev. Lett. **80**, 890 (1998).
[21] C. Adamo and V. Barone, The Journal of Chemical Physics **108**, 664 (1998), https://pubs.aip.org/aip/jcp/article-pdf/108/2/664/10791395/664_1_online.pdf.
[22] B. Hammer, L. B. Hansen, and J. K. Nørskov, Physical Review B **59**, 7413 (1999).
[23] E. E. Dahlke and D. G. Truhlar, The Journal of Physical Chemistry B **109**, 15677 (2005), pMID: 16852988, https://doi.org/10.1021/jp052436c.
[24] N. E. Schultz, Y. Zhao, and D. G. Truhlar, The Journal of Physical Chemistry A **109**, 11127 (2005), pMID: 16331896, https://doi.org/10.1021/jp0539223.
[25] Y. Zhao and D. G. Truhlar, The Journal of Chemical Physics **128**, 184109 (2008), https://pubs.aip.org/aip/jcp/article-pdf/doi/10.1063/1.2912068/15412612/184109_1_online.pdf.
[26] J. P. Perdew, A. Ruzsinszky, G. I. Csonka, O. A. Vydrov, G. E. Scuseria, L. A. Constantin, X. Zhou, and K. Burke, Phys. Rev. Lett. **100**, 136406 (2008).
[27] R. Peverati, Y. Zhao, and D. G. Truhlar, The Journal of Physical Chemistry Letters **2**, 1991 (2011), https://doi.org/10.1021/jz200616w.
[28] R. Peverati and D. G. Truhlar, Journal of Chemical Theory and Computation **8**, 2310 (2012), pMID: 26588964, https://doi.org/10.1021/ct3002656.
[29] J. P. Perdew, S. Kurth, A. c. v. Zupan, and P. Blaha, Physical Review Letters **82**, 2544 (1999).
[30] J. Tao, J. P. Perdew, V. N. Staroverov, and G. E. Scuseria, Phys. Rev. Lett. **91**, 146401 (2003).

[31] J. P. Perdew, J. Tao, V. N. Staroverov, and G. E. Scuseria, The Journal of Chemical Physics **120**, 6898 (2004), https://pubs.aip.org/aip/jcp/article-pdf/120/15/6898/10855090/6898_1_online.pdf.

[32] Y. Zhao and D. G. Truhlar, The Journal of Chemical Physics **125**, 194101 (2006), https://pubs.aip.org/aip/jcp/article-pdf/doi/10.1063/1.2370993/15391406/194101_1_online.pdf.

[33] Y. Zhao and D. G. Truhlar, Theoretical Chemistry Accounts **120**, 215 (2008).

[34] J. P. Perdew, A. Ruzsinszky, G. I. Csonka, L. A. Constantin, and J. Sun, Phys. Rev. Lett. **103**, 026403 (2009).

[35] J. P. Perdew, A. Ruzsinszky, G. I. Csonka, L. A. Constantin, and J. Sun, Phys. Rev. Lett. **106**, 179902 (2011).

[36] R. Peverati and D. G. Truhlar, The Journal of Physical Chemistry Letters **3**, 117 (2012), https://doi.org/10.1021/jz201525m.

[37] R. Peverati and D. G. Truhlar, Phys. Chem. Chem. Phys. **14**, 13171 (2012).

[38] J. Sun, B. Xiao, and A. Ruzsinszky, The Journal of Chemical Physics **137**, 051101 (2012), https://pubs.aip.org/aip/jcp/article-pdf/doi/10.1063/1.4742312/15452686/051101_1_online.pdf.

[39] J. Sun, R. Haunschild, B. Xiao, I. W. Bulik, G. E. Scuseria, and J. P. Perdew, The Journal of Chemical Physics **138**, 044113 (2013), https://pubs.aip.org/aip/jcp/article-pdf/doi/10.1063/1.4789414/15459289/044113_1_online.pdf.

[40] J. Sun, J. P. Perdew, and A. Ruzsinszky, Proceedings of the National Academy of Sciences **112**, 685 (2015), https://www.pnas.org/doi/pdf/10.1073/pnas.1423145112.

[41] J. Sun, A. Ruzsinszky, and J. P. Perdew, Phys. Rev. Lett. **115**, 036402 (2015).

[42] P. J. Stephens, F. J. Devlin, C. F. Chabalowski, and M. J. Frisch, The Journal of Physical Chemistry **98**, 11623 (1994), https://doi.org/10.1021/j100096a001.

[43] A. D. Becke, The Journal of Chemical Physics **98**, 5648 (1993), https://pubs.aip.org/aip/jcp/article-pdf/98/7/5648/11091662/5648_1_online.pdf.

[44] F. A. Hamprecht, A. J. Cohen, D. J. Tozer, and N. C. Handy, The Journal of Chemical Physics **109**, 6264 (1998), https://pubs.aip.org/aip/jcp/article-pdf/109/15/6264/10793650/6264_1_online.pdf.

[45] C. Adamo and V. Barone, The Journal of Chemical Physics **110**, 6158 (1999), https://pubs.aip.org/aip/jcp/article-pdf/110/13/6158/10797469/6158_1_online.pdf.

[46] M. Ernzerhof and G. E. Scuseria, The Journal of Chemical Physics **110**, 5029 (1999), https://pubs.aip.org/aip/jcp/article-pdf/110/11/5029/10797375/5029_1_online.pdf.

[47] P. J. Wilson, T. J. Bradley, and D. J. Tozer, The Journal of Chemical Physics **115**, 9233 (2001), https://pubs.aip.org/aip/jcp/article-pdf/115/20/9233/10835630/9233_1_online.pdf.

[48] W.-M. Hoe, A. J. Cohen, and N. C. Handy, Chemical Physics Letters **341**, 319 (2001).

[49] A. J. COHEN and N. C. HANDY, Molecular Physics **99**, 607 (2001), https://doi.org/10.1080/00268970010023435.

[50] T. Yanai, D. P. Tew, and N. C. Handy, Chemical Physics Letters **393**, 51 (2004).

[51] T. W. Keal and D. J. Tozer, The Journal of Chemical Physics **123**, 121103 (2005), https://pubs.aip.org/aip/jcp/article-pdf/doi/10.1063/1.2061227/15372632/121103_1_online.pdf.

[52] Y. Zhao, N. E. Schultz, and D. G. Truhlar, The Journal of Chemical Physics **123**, 161103 (2005), https://pubs.aip.org/aip/jcp/article-pdf/doi/10.1063/1.2126975/15373556/161103_1_online.pdf.

[53] J. Heyd, G. E. Scuseria, and M. Ernzerhof, The Journal of Chemical Physics **118**, 8207 (2003), https://pubs.aip.org/aip/jcp/article-pdf/118/18/8207/10847843/8207_1_online.pdf.

[54] J. Heyd, G. E. Scuseria, and M. Ernzerhof, The Journal of Chemical Physics **124**, 219906 (2006), https://pubs.aip.org/aip/jcp/article-pdf/doi/10.1063/1.2204597/15387022/219906_1_online.pdf.

[55] A. V. Krukau, O. A. Vydrov, A. F. Izmaylov, and G. E. Scuseria, The Journal of Chemical Physics **125**, 224106 (2006), https://pubs.aip.org/aip/jcp/article-pdf/doi/10.1063/1.2404663/13263224/224106_1_online.pdf.

[56] Y. Zhao and D. G. Truhlar, The Journal of Physical Chemistry A **110**, 13126 (2006), pMID: 17149824, https://doi.org/10.1021/jp066479k.

[57] Y. Zhao and D. G. Truhlar, Journal of Chemical Theory and Computation **4**, 1849 (2008), pMID: 26620329, https://doi.org/10.1021/ct800246v.

[58] M. Renz, K. Theilacker, C. Lambert, and M. Kaupp, Journal of the American Chemical Society **131**, 16292 (2009), pMID: 19831383, https://doi.org/10.1021/ja9070859.

[59] M. Kaupp, M. Renz, M. Parthey, M. Stolte, F. Würthner, and C. Lambert, Phys. Chem. Chem. Phys. **13**, 16973 (2011).

[60] R. Peverati and D. G. Truhlar, The Journal of Physical Chemistry Letters **2**, 2810 (2011), https://doi.org/10.1021/jz201170d.

[61] A. Austin, G. A. Petersson, M. J. Frisch, F. J. Dobek, G. Scalmani, and K. Throssell, Journal of Chemical Theory and Computation **8**, 4989 (2012), pMID: 26593191, https://doi.org/10.1021/ct300778e.

[62] R. Peverati and D. G. Truhlar, Phys. Chem. Chem. Phys. **14**, 16187 (2012).

[63] L. Lu, H. Hu, H. Hou, and B. Wang, Computational and Theoretical Chemistry **1015**, 64 (2013).

[64] P. D. Mezei, G. I. Csonka, and M. Kállay, Journal of Chemical Theory and Computation **13**, 4753–4764 (2017).
